# PolyQ Database—an integrated database on polyglutamine diseases

**DOI:** 10.1093/database/baad060

**Published:** 2023-08-18

**Authors:** Bernardo Estevam, Carlos A Matos, Clévio Nóbrega

**Affiliations:** ABC-Ri, Algarve Biomedical Center Research Institute, Campus de Gambelas, Faro 8005-139, Portugal; Faculdade de Medicina e Ciências Biomédicas, Universidade do Algarve, Campus de Gambelas, Faro 8005-139, Portugal; ABC-Ri, Algarve Biomedical Center Research Institute, Campus de Gambelas, Faro 8005-139, Portugal; Faculdade de Medicina e Ciências Biomédicas, Universidade do Algarve, Campus de Gambelas, Faro 8005-139, Portugal; ABC-Ri, Algarve Biomedical Center Research Institute, Campus de Gambelas, Faro 8005-139, Portugal; Faculdade de Medicina e Ciências Biomédicas, Universidade do Algarve, Campus de Gambelas, Faro 8005-139, Portugal

## Abstract

Polyglutamine (polyQ) diseases are neurodegenerative disorders caused by abnormally expanded Cytosine, Adenine, Guanine (CAG) triplet repeat sequences in the coding region of otherwise unrelated genes. Until now, nine different polyQ diseases have been described: Huntington’s disease, dentatorubral-pallidoluysian atrophy, spinal and bulbar muscular atrophy and six types of spinocerebellar ataxias—1, 2, 3, 6, 7 and 17. The pathogenic expansion translates into an aberrant tract of glutamines in the encoded proteins, compromising several cellular functions and biological processes. There is currently no cure available for the progressive neurodegenerative disorders caused by the ensuing cytotoxic alterations. Although each disease is considered rare, polyQ diseases constitute the largest group of monogenic neurodegenerative disorders. Information about these disorders is scattered among several books, articles and general databases, hindering exploration by students and researchers, but also by patients and their families. Therefore, we aimed to develop a free online database to fill this gap, by centralizing relevant available information. The PolyQ Database is a platform that focuses on all nine polyQ diseases and offers information about topics that are pertinent for scientists, clinicians and the general public, including epidemiology, the characteristics of the causative genes and the codified proteins, the pathophysiology of the diseases and the main clinical manifestations. The database is available at https://polyq.pt/, and it is the first of its kind, focusing exclusively on this group of rare diseases. The database was conceived to be continuously updated and allow incorporation and dissemination of the latest information on polyQ diseases.

## Introduction

Polyglutamine (polyQ) diseases are a group of nine neurodegenerative diseases caused by abnormal expansion of CAG triplet sequences in the coding regions of nine unrelated genes. These sequences are polymorphic in the human population and are translated as a polyQ tract within the encoded proteins (1). PolyQ diseases are genetically inherited, and currently the diseases that comprise the group are: Huntington’s disease (HD), dentatorubral-pallidoluysian atrophy, spinal and bulbar muscular atrophy and the spinocerebellar ataxias (SCAs) types 1, 2, 3, 6, 7 and 17 (2, 3). The threshold number of repetitions that renders genes pathological varies across the different polyQ diseases. As example, HD manifests in carriers of 39 or more repeats (4), whereas SCA3, also known as Machado-Joseph disease (MJD/SCA3), manifests when the repetitions are 60 or more (5).

Disease manifestations observed in each disease reflect the functional compromise of specific regions of the central nervous system that undergo degeneration. For example, all six polyQ SCAs involve ataxia as a consequence of cerebellar degeneration, while chorea is observed in HD, resulting from basal ganglia function decline (2, 6, 7). More severe phenotypes and early disease onset are generally associated with longer repeat expansions (8, 9). To date, there are no disease-modifying treatments for any of the polyQ diseases, and patients, who rely solely on symptomatic treatment, tend to die within 10–15 years after disease onset (10).

A database is a collection of structured information or data that is typically stored electronically in a computer system. A database management system (DBMS) is a software tool that enables users to create and manage databases. It includes a set of programs that allow users to create, protect, read, update and delete the contents within a database. Accordingly, every database has its own DBMS, which is used to manage the database and ensure its proper functioning.

We aimed to design and setup a new database, the PolyQ Database, which collected and centralized information on pre-defined topics for all nine polyQ diseases. The produced database organizes the information in a concise manner, with original images and schemes, as a tool for researchers, students, patients and their families. The final goal was for the database to be available online, with free access to anyone, therefore creating a new global resource with extended information about polyQ disorders and amenable to continuous updating. The database is accessible at https://polyq.pt/.

## Materials and methods

### Selection of sections for the database

The selection of the sections to be included in the database took into consideration several factors. First, it was essential to provide information on all nine polyQ diseases that have been described so far. Second, it was important to have sections on pathology topics that would be of interest to a broad audience, including clinicians, patients, their families and the general public, such as information on disease signs and symptoms and epidemiologic data. Third, it was also important to include details on issues that would appeal to scientific researchers in the field, including information concerning the main features of the genes and the proteins associated with each disease. Finally, it was also reasoned that the same sections should be presented for all diseases included in the database. A full structure of all details and sections was envisioned (Supplementary Figure S1), although not all sections are included in the current version of the database.

### Search for information

After deciding on the sections to include in the database, the information to fill in those sections was searched and manually curated from several online sources, along with theme-related books. Most of the information was obtained from published papers, which were found in public databases, such as the National Center for Biotechnology Information (NCBI), ResearchGate, ScienceDirect and Wiley Online Library, and from two very focused important databases: Uniprot and GenomeBrowser. All the sources that were consulted are referenced throughout the PolyQ Database.

To retrieve general information about all polyQ diseases we used GeneReviews as the first source. GeneReviews is indexed in PubMed, being written by experts in the area. GeneReviews goes through a rigorous editing and peer review process, which makes it a relevant and impactful source.

We also used as a reference the book Polyglutamine Disorders by Clévio Nóbrega and Luís Pereira de Almeida. Polyglutamine Disorders book curates and merges information from recent discoveries and experts in this research area. The book presents molecular and cellular mechanisms for polyQ diseases. Additionally, it also has information regarding the genetic aspects, clinical findings and disease presentation.

We also performed Google searches to retrieve some information missing in the previous sources or try to elucidate some contradictory information. Nevertheless, the information used in these searches was always from trustful sources, such as papers or books.

PubMed, ResearchGate, ScienceDirect and Wiley Online Library databases were also used to find the information needed considering the most recent and impactful articles. The search was mostly done using the disease name and the operator ‘AND’ followed by the type of information needed, such as epidemiology, clinical manifestations, neuropathology, gene affected or protein, among others. The selection of the studies from these searches was made considering the most recent studies and, in some cases, considering the impact factor of the journal.

Although gene and protein information was found in the previous sources, most of the information used was retrieved from Genome Browser by the University of California Santa Cruz and Uniprot, respectively. In all disease pages, gene location, number of exons and introns, codified length and full length were retrieved from Genome Browser. Protein biological and molecular functions, length, structure and domains were mainly found and used from Uniprot.

### Development tools and implementation

The PolyQ Database was created using programming languages for web development. For the platform back-end, Django, a python library and a Microsoft SQL server were used. Hypertext markup language, cascading style sheets and JavaScript were used for the front-end. The deployment of the platform was done in a virtual private server running on Ubuntu (on Linux operating system).

## Results

### Platform layout and structure

**Figure 1.**
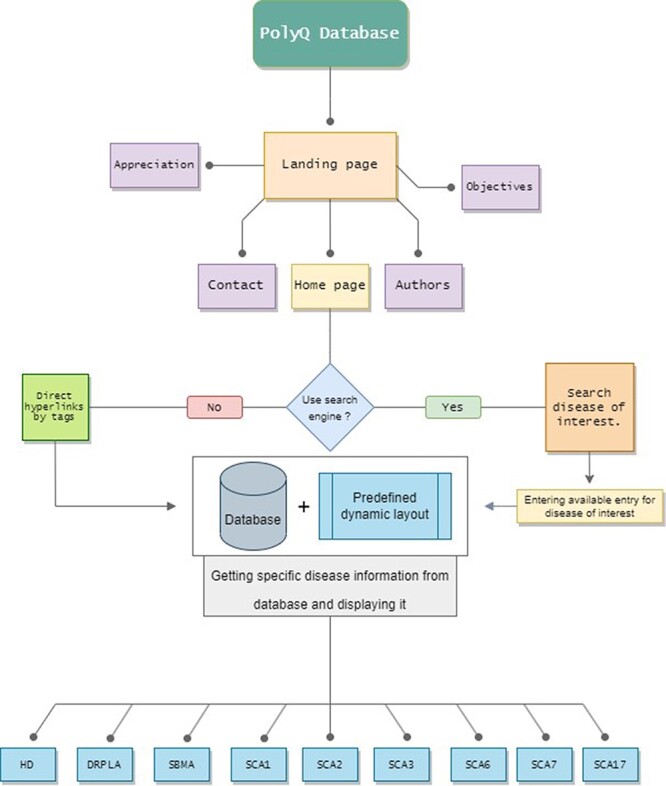
The PolyQ Database structure, depicting all the available pages, routes and disease options. Home page has two different options to access any Disease page.

The platform layout and structure were developed to allow rapid and user-friendly use of the database (Figure 1). One of the main aims of the developed interface was to be easy to browse with all the options at the disposal of the visitor and with a responsive layout for every device that the database is visited on. The final structure of the PolyQ Database comprises an initial Landing page, which can redirect the visitor to a Contact page, an Appreciation page, an Authors page, an Objectives page and the Home page. The Contact page enables communication with the database developers. The objectives of the PolyQ Database are presented in the Objectives page and several online resources are mentioned in the Appreciation page, as they were instrumental in the development of the database. On the Home page, visitors can either search for the disease of interest or simply scroll down and select one of the available diseases by tags, this way being redirected to the selected Disease page.

### Disease pages content

All the Disease pages presented in the PolyQ Database have the same sections and type of information. More precisely, they include an introduction, containing a brief preliminary account on the disease and information on inheritance features. The next section focuses on the first descriptions of that disease, with the first historical reports on each disorder, with authors properly referenced. Then, there is a section with epidemiology data, with the most important epidemiological features (around the globe) of every disease. An original map summarizing the information found in several different articles is presented, conveying the information in a simplified and illustrative form. The following sections describe the causative gene and the protein associated with each disorder. Information such as the name of the gene, its chromosomal location, how and where the gene is mutated and different degrees of mutation are all presented, along with a simple representation of the structure of the gene. Information on the protein includes its domain structure, protein functions and subcellular localization. The next section provides information about the most important pathophysiological mechanisms that have been described to be involved in disease causation and progression. As a supplement to the text, an image is displayed that translates the textual information into a scheme. Next, there is a section describing the main clinical manifestations of each disease, along with relevant neuropathological findings. Finally, in the last section, we included the active clinical trials for the disease, according to ClinicalTrials.gov.

### Additional features of the disease pages

**Figure 2.**
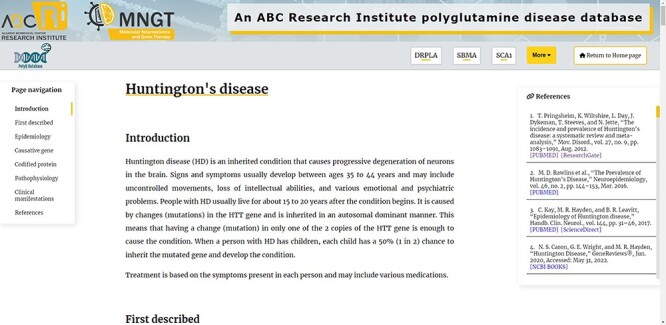
Top of the Huntington’s Disease page, presenting all the additional features of the Diseases pages mentioned in the text.

When loading a Disease page, there are some features that help navigation and improve interactivity (Figure 2). Visitors can change between Disease pages easily on the top navigation bar and can also return to the Home page (Figure 2). There is a page navigation tool that scrolls the page to the clicked topic (Figure 2). In the text, there are several references that can be easily analysed with the help of the references side bar, presenting the authors of the respective bibliographical sources and direct links to the respective article, book or other types of reference (Figure 2).

Besides the above mentioned features, there is on the right bottom corner of the website a form icon where users can click and fill out a feedback form, sending it directly and automatically to authors. This form appears in all disease pages, as these pages have content with the information that requires constant curation and update. The form requires a name, subject and a message, while email is optional. The form facilitates the interaction and feedback of the database users with database authors ; nevertheless, users can also contact us directly via the email presented on our contact page.

## Conclusions

### Utility of the database for researchers, clinicians and patients

As mentioned, the rationale behind the development of polyQ database was to create a useful resource for a broad audience. In this sense, the database will be useful for researchers, as it constitutes a centralized and updated repository of information about these diseases. Moreover, it allows a simple and straightforward comparison of the different polyQ diseases and a platform for mining data, integrating findings and generating new hypotheses. Finally, it offers collaboration opportunities, as researchers can identify other scientists working on similar topics. For clinicians, the polyQ database provides state of the art information on these rare diseases, which might not yet be available in other resources. For patients, the database provides a resource with information about the disease and importantly on the clinical trials that are actively recruiting. In the future, we also aim to link the database to patients’ disease associations worldwide and to make available different resources and educational materials that can be used by patients and their families to raise awareness.

Overall, the PolyQ database serves as a centralized hub for information on polyQ diseases, providing comprehensive and up-to-date information, thus facilitating collaboration, supporting clinical decision-making and empowering patients in their journey of understanding and managing these diseases.

### Uniqueness and importance of the PolyQ Database

The PolyQ Database is the first online resource that specifically collects and centralizes information on polyQ diseases. While other databases, such as OMIM or Orphanet, also have information on these disorders, they are general disease databases. Moreover, the PolyQ Database aims to serve as a centralized repository of polyQ disease information for any type of interested visitor. Additionally, the PolyQ database gives a strong emphasis on molecular and genetic aspects of polyQ diseases. It provides detailed information on the underlying genetic mutations, the expansion of polyQ repeats and their impact on protein structure and function. Compared to OMIM and Orphanet, the PolyQ database is also more flexible to update and reformulate. Being dedicated to a small number of diseases, it is easier to introduce a new section with information that is available to all diseases. Finally, the fact that PolyQ Database has a very simple and user-friendly interface allows wider access to the information for everyone, contributing to that end. The easiness of use of the database was informally tested among researchers from the authors’ laboratory and research centre.

### Future updates of the platform

The platform has the possibility of being easily updated as new information arises in the field of PolyQ diseases research. Besides the addition of new sections and improved content, the database will be frequently curated for errors and to introduce new information about the existing sections, especially based on feedback from users. Apart from this, additional features are already scheduled to be implemented, to reach a wider audience and improve user interaction. Within the first 6 months after the publication of the article describing the database, two language options will be added: Spanish and Portuguese. More language support will be added, in time, depending on the feedback obtained. In a year, other updates will focus on improving information on the genes and proteins. In due time, the database will offer a more interactive and illustrative scheme with condensed data. Also, new sections of general interest will be added. These will be chosen according to users’ feedback and new topics of interest that may arise from research. Finally, in 1–2 years span, we plan to enhance the navigation system in the back end of the software/website to make it easier for maintenance personnel to understand the logic behind it. This improvement is aimed at preventing any difficulties that may arise during maintenance tasks, ensuring that the system’s back-end navigation is efficient without requiring extensive knowledge or expertise.

### The PolyQ Database limitations

As with any resource, the PolyQ Database has some limitations that we hope to attenuate and mitigate during the improvement of the database. One possible limitation is the selection and publication bias, as we selected the information based on searches and in our knowledge in this research area. Therefore, some publications and information about polyQ diseases might be missing. Another possible limitation is related to data availability and completeness. PolyQ diseases are rare; however, among them, some are even rarer. Therefore, information and data for these diseases are limited, as compared to other more common diseases within the group. Finally, a limitation of the database is related to user interpretation and language bias. The database’s effectiveness is also dependent on the users’ knowledge and ability to interpret the information provided. Additionally, the database is only in English, which limits its accessibility.

## Supplementary Material

baad060_Supp

## Data Availability

PolyQ Database is available at https://polyq.pt/.
