# Supplementary figures and images for: PolyQ Database—an integrated database on polyglutamine diseases
Source: Database (Oxford). 2023 Aug 18;2023:baad060. doi: 10.1093/database/baad060 (PMC10440501; doi:10.1093/database/baad060)

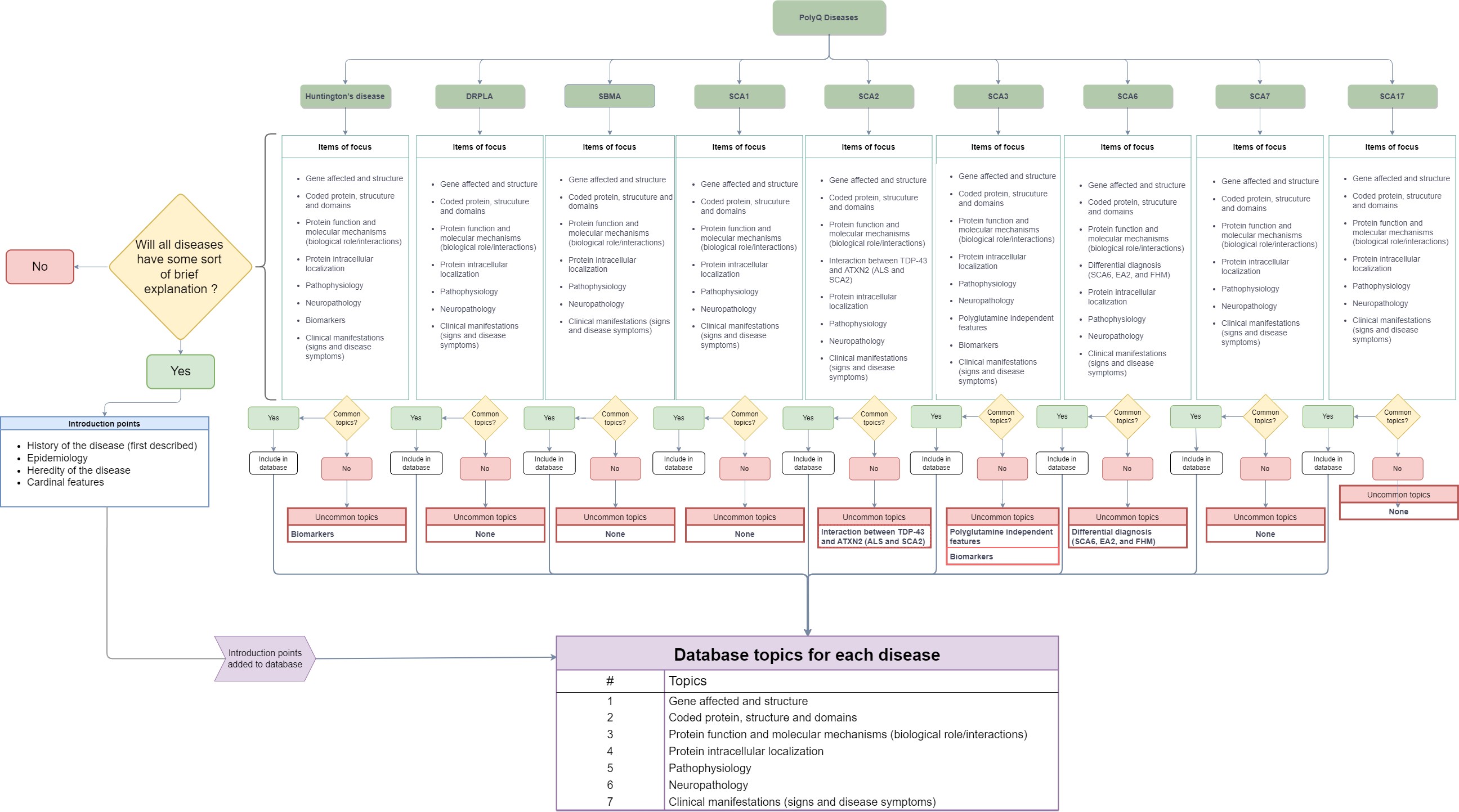

Supplement: baad060_Supp [file baad060_supp.zip › suppl_data/Sup1.jpg]
